# EFFECT OF VISION AND HEARING IMPAIRMENT ON LIFE SPACE MOBILITY AMONG OLDER MEXICAN-AMERICANS

**DOI:** 10.1093/geroni/igz038.3271

**Published:** 2019-11-08

**Authors:** Viridiana Saenz Monsivais, Brian Downer, Mukaila Raji, Yong-Fang Kuo

**Affiliations:** 1 University of Texas Medical Branch, Galveston, Texas, United States; 2 University of Texas Medical Branch, Galveston, TX, Galveston, Texas, United States

## Abstract

Background: Poor vision and hearing can restrict an older adult’s life space mobility (LSM). Our objective was to examine if poor vision and hearing differentially impact the decline in LSM over a 2-year period among older Mexican-Americans. Methods: Data came from waves 7 (2010/11) and 8 (2012/13) of the Hispanic EPESE. Our final sample included 452 participants aged >79 years. Participants who said they could not recognize a person across the street, room, or arms-length were classified as having poor vision. Participants who said they could not understand a normal voice in a quiet room were classified as having poor hearing. Participants who decreased >10 points on the Life-Space Assessment were classified as having a decline in LSM. Logistic regression was used to estimate the odds for a decline in LSM according to poor hearing and vision, controlling for baseline demographic and health characteristics. Results: Poor vision was associated with 2.70 (95% CI=1.37-5.62) greater odds for a decline in LSM. This association varied according to depressive symptoms. Poor vision was significantly associated with a decline in LSM for participants without high depressive symptoms (OR=5.04, 95% CI=2.00-15.5), but not for participants with depression (OR=0.63, 95% CI=0.12-3.13). The association between poor hearing and decline in LSM was not significant (OR=0.68, 95% CI=0.36-1.27). Conclusions: Poor vision is a risk factor for decline in LSM, especially for older Mexican-Americans who do not have high depressive symptoms. Poor hearing does not appear to be a risk factor for a decline in LSM for older Mexican-Americans.

